# Anthropogenic Nest Cavities Used by Snow Buntings in an Urban Arctic Landscape

**DOI:** 10.1002/ece3.71457

**Published:** 2025-05-23

**Authors:** Samuelle Simard‐Provençal, Patricia Rokitnicki, Rebecca Golat, François Vézina, Oliver P. Love, Emily A. McKinnon

**Affiliations:** ^1^ Department of Integrative Biology University of Windsor Windsor Ontario Canada; ^2^ Département de Biologie, Chimie et Géographie Université du Québec à Rimouski Rimouski Québec Canada; ^3^ Access Program University of Manitoba Winnipeg Manitoba Canada

**Keywords:** Arctic, climate change, songbird, synanthropic, urban ecology

## Abstract

Northern and Arctic ecosystems are experiencing rapid climate change, and simultaneously, human populations in the North are growing and centralizing. The Snow Bunting ( *Plectrophenax nivalis* ) is a Holarctic‐breeding songbird and abundant in urban Iqaluit (pop. 7400), Nunavut. In nonurban areas of the Arctic, nest cavities are a limited resource for breeding Snow Buntings. Our goal was to assess the extent of Snow Buntings' use of anthropogenic structures versus natural rock cavities for nesting in Iqaluit. We found 160 Snow Bunting nests (2023, 2024) in Iqaluit; 45% of these were in anthropogenic nest cavities, for example, in vents in buildings or human‐made rock structures (e.g., revetment gabions). This is the first documentation of extensive anthropogenic cavity use of Snow Buntings in an urban‐Arctic environment. Nests in anthropogenic structures were significantly higher off the ground than nests in natural cavities but were similar in orientation and depth. Natural cavities were exclusively in rock. Anthropogenic nesting cavities were also primarily in rock (77%) but about 10% of cavities were in other materials, including wood, metal, or buildings. Given this flexibility in nest cavity use, Snow Buntings may be less limited for nest cavities in the urban environment compared to a natural landscape, although the impacts of anthropogenic nest cavities on reproductive success remain to be explored.

## Graphical abstract

In this study, we document extensive use of anthropogenic structures for nesting by a holarctic‐breeding songbird, the Snow Bunting, in the rapidly urbanizing town of Iqaluit, Nunavut. Snow Buntings typically nest in natural rock crevices, but in town they nested in buildings, human‐constructed rock piles, vents, and even metal pipes. These human‐created structures have different characteristics compared with natural nest cavities, and may impact the reproductive success of this declining species.

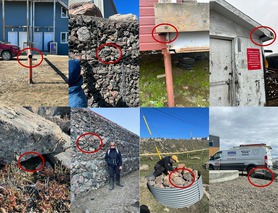

## 1 Introduction

Urbanized environments can provide benefits and costs for wildlife, including birds (Marzluff 2001). Birds in urban areas may nest earlier but have lower productivity (Chamberlain et al. 2009). Urban areas support fewer predators of birds and bird nests (Eötvös et al. 2018), and human‐built structures can provide nest cavities (Tomasevic and Marzluff 2017), especially in cities where natural nest cavities have been removed by people (Sandoval et al. 2021). Birds can be attracted to urban areas where they have abundant resources, including nest cavities, which can lead to high nesting densities with no apparent productivity cost (Rodewald and Shustack 2008) There has been no research on cavity‐nesting birds in Arctic cities, such as Iqaluit (ᐃᖃᓗᐃᑦ), the largest and capital city of Nunavut, Canada, and the largest city above the treeline in North America (population ~7400, Statistics Canada 2023). Cavity‐nesting songbirds in Iqaluit have access to both anthropogenic and natural nesting sites. Human‐built structures of rock, wood, plastic, or metal can all serve as nest cavities, whereas natural cavities are almost exclusively rock.

Snow Buntings (Inuktitut: *qupanuak*, Latin: *Plectrophenax nivalis* ) frequently nest in and around human settlements throughout their Arctic and sub‐Arctic breeding range (Montgomerie and Lyon 2020), and are abundant in the city of Iqaluit, Nunavut (63.74° N, −68.52° W; all authors, pers. obs). The goal of this study was to document the extent of Snow Buntings' use of anthropogenic versus natural nesting cavities within the city of Iqaluit. Snow Buntings naturally nest in rock cavities and cracks, under boulders, and on cliff faces and use nest boxes, oil drums, stone and concrete foundations, or even animal skulls as nest sites (Montgomerie and Lyon 2020). One source describes a traditional practice of Inuit to purposefully create nesting habitat for Snow Buntings by piling up rocks; this tradition continues today in the construction (and maintenance) of wooden nest boxes in some communities in Alaska, USA (Keim 2022). Based on this history of anthropogenic interactions and the flexible nesting habits of this species, we expected that buntings in Iqaluit would make use of the abundance of human‐built structures, including buildings, industrial structures, and human‐built rock revetments.

## 2 Methods

### 2.1 Study Site

**FIGURE 1:**
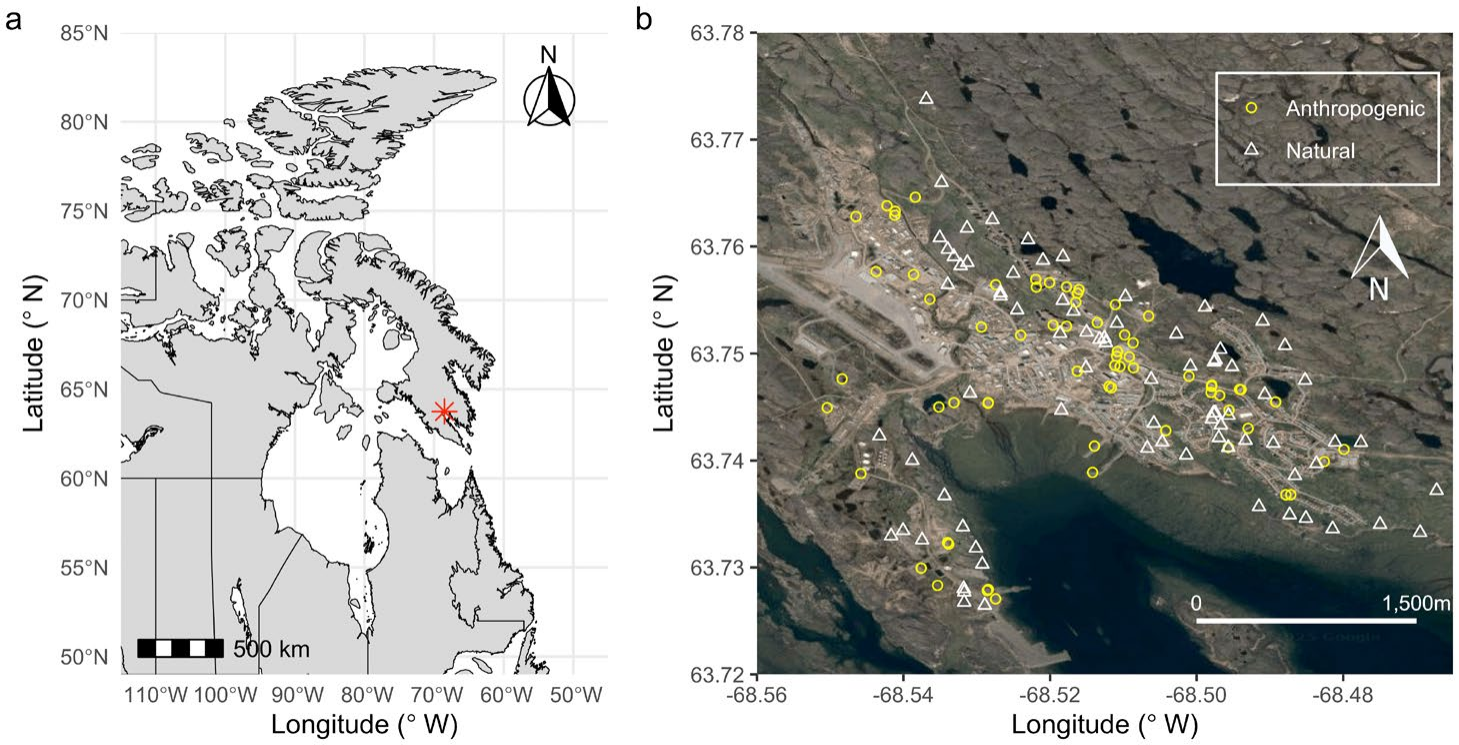
(a) Map of Iqaluit at large scale (red star; note that scale bar is approximate due to projection distortion at high latitudes) and (b) nest locations within Iqaluit, with nest type indicated as circle for anthropogenic and triangle for natural cavities. Satellite map for panel b from GoogleEarth, made with *ggmap* (Kahle and Wickham 2013).

This research was conducted in the City of Iqaluit ᐃᖃᓗᐃᑦ, NU, Canada (63.74° N, −68.52° W; Figure 1), in the adjacent suburb of Niaqunngut (Apex, 63.73° N, 68.45° W). The surrounding land, water, and ice are covered by the Nunavut Agreement and are within the Inuit Nunangat. All work was supported by permits from the Government of Nunavut (2023‐048), Environment and Climate Change Canada (10808), and the University of Windsor Animal Care Committee (AUPP‐22‐04). Iqaluit has experienced rapid population growth and infrastructure development since becoming the territorial capital in 1999. The population increased from 2050 people to 7429 people over the last 50 years (Statistics Canada 2023), leading to increased disturbance and infrastructure, including a new international airport completed in 2017. Impacts of this rapid population growth and development on local bird diversity have not been studied.

### 2.2 Nest Searching

Nest searching was conducted daily by a core team of experienced ornithologists between June 1 and July 18 in 2023 and 2024. The team focused on following adults to the nest, not on searching for specific cavity types, therefore avoiding preferential detection of either artificial or natural nest cavities. The team made careful observations of any buntings seen, paying particular attention to females. Once a female was located, her behavior (e.g., carrying nesting materials or arthropods) provided insight into the stage of nest development (i.e., nest building, incubation, or chick rearing). Nests were confirmed active by visual inspection or with the use of an endoscope camera (Mastercraft 3.3‐ft Cable Digital Inspection Camera with 2.7‐in LCD). Once a nest site was identified, GPS coordinates were recorded in the field using the Gaia GPS mobile app, and waypoints were visually confirmed by examining location relative to buildings and other features visible on the satellite map.

For each nest, we recorded the type of nest cavity (natural or anthropogenic), and the substrate that formed the nest cavity, and classified substrates into: rock, metal, wood, or buildings, which consisted of a mixture of material. In these cases the external material around the nest entrance was often different than the material where the nest was placed inside the cavity, for example, a plastic vent cover with an interior made of wood. We also recorded the distance of the nearest edge of the nest from the cavity entrance (nest cavity depth), the height of the nest cavity entrance off the ground surface immediately outside the nest and the directional bearing (relative to magnetic North) of the main entrance to the cavity facing outwards. Occasionally nests had multiple entrances/exits so we measured the entrance that we visually observed birds using most frequently. In the case of rock‐revetments where birds could fly out of multiple gaps between rocks, all the “exits” were facing in the same direction, perpendicular to the rock revetment. We considered nests in rock piles and caged‐rock revetments (gabions) created by humans as “artificial” as these piles were often constructed around buildings and consisted of much looser piles of uniform rocks, compared with natural rock cavities or natural boulder piles. Natural rock cavities were in bedrock cracks, piles of boulders or natural scree slopes. All nest site measurements were taken to the nearest centimeter and nearest degree.

### 2.3 Descriptive Analysis

We compared the locations of nests in both cavity types to assess if nests showed any spatial associations or were randomly dispersed by calculating the cross‐type Ripley's K‐function, with statistical significance evaluated using a Monte Carlo simulation envelope (999 permutations; Baddeley et al. 2015; Baddeley and Turner 2005). We used Welch's *t*‐tests to compare characteristics of artificial and natural nest cavities (height off ground, depth) and compared bearing of nest cavity entrance by type using R package “circular” (Agostinelli and Lund 2024). We used a chi‐squared test of equal proportions to compare the proportion of nests above ground for anthropogenic and natural nests. All data were explored to assess if they met assumptions of statistical tests used. We chose Welsh' *t‐*test to account for heteroscedasticity in nest height by nest type, and we used the Mardia–Watson–Wheeler circular test for differences in bearing by nest type, as the data were highly dispersed (concentration estimate < 2, Agostinelli and Lund 2024). All statistical analysis and mapping were conducted in R Version 4.4.3 (R Core Team 2025).

## 3 Results

**FIGURE 2:**
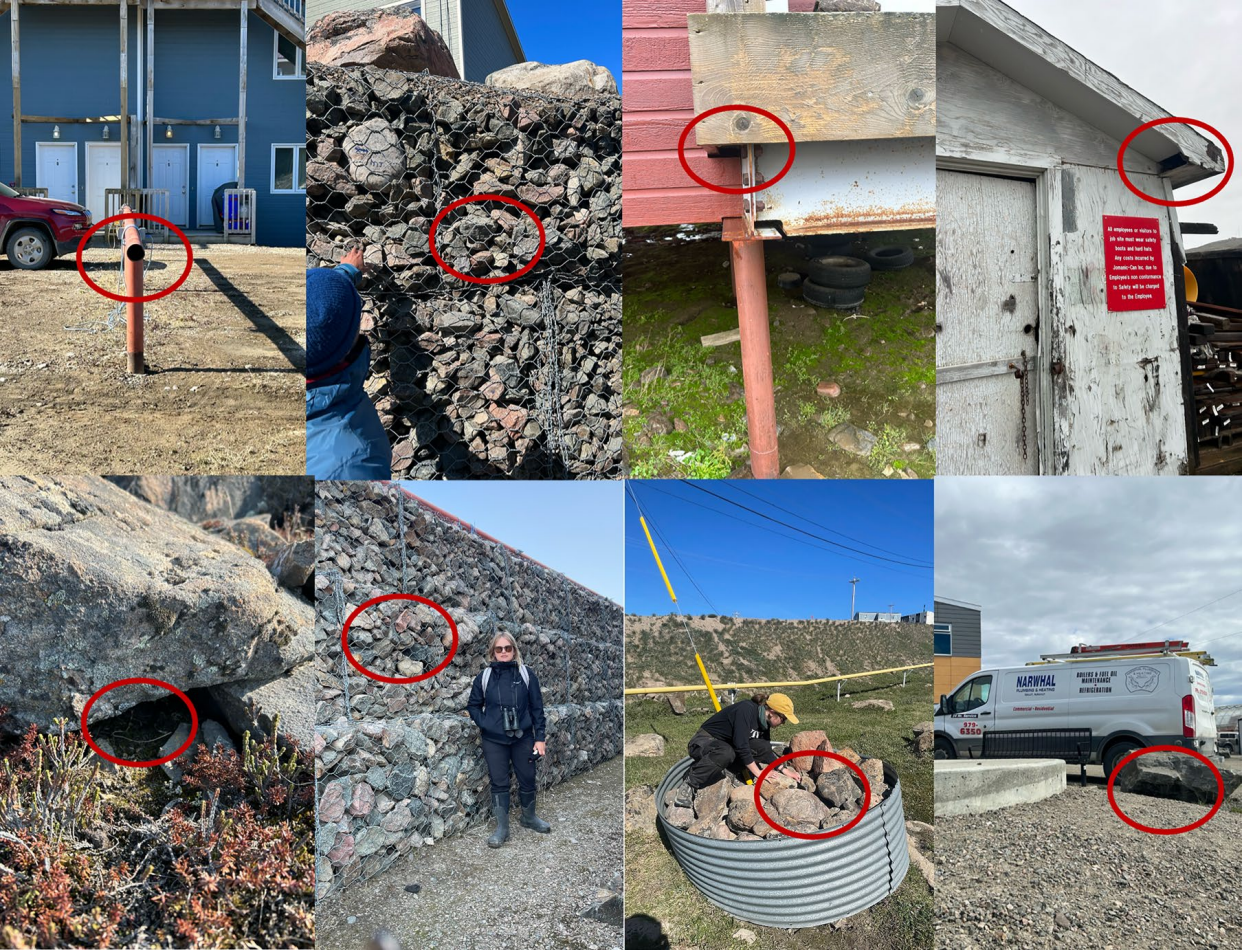
Examples of nest sites (circled in red) in anthropogenic cavities and one natural cavity (bottom left). Top row (L–R): Nest in a metal pipe, in a rock gabion, two in buildings; bottom row (L–R): Nest in a natural cavity under a natural rock, in a gabion wall, in a cable anchoring site, and under a boulder place to define a driveway. Pictures taken by field team members S. Simard‐Provençal, P. Rokitnicki, and E. McKinnon.

We collected information on 160 Snow Buntings nests (61 in 2023, 99 in 2024). Of these, 71 were located in artificial substrate of various types (Figure 2) and 89 were in natural rock cavities (Table 1). Nests in both substrate types were interspersed throughout the urban landscape (Figure 1). To check for clustering of nest types, we plotted the observed Kcross function versus the expected pattern with no clustering (e.g., complete spatial randomness). We found that the observed Kcross function tended to be above the expected function, indicating that anthropogenic and natural nests were less likely to occur near one another than would be expected by chance. We further explored spatial associations between anthropogenic and natural nest locations using a Monte Carlo simulation with 999 permutations. The observed Kcross function approached the lower bound of the 95% simulation envelope at distances of ~200 m, suggesting a weak tendency for spatial segregation at this scale. However, the function remained within the simulation envelope at all other distances, indicating no significant spatial interaction between nest types across the broader study area.

**TABLE 1:** Nest substrate and characteristics of 160 Snow Bunting nests in Iqaluit, NU.

Cavity type	Nest substrate	Mean cavity height (min, max)	Mean depth of nest (min, max)	Number of nests
Anthropogenic	Buildings (mixed material)	77 cm (0–154)	15.5 cm (12–19)	4
	Metal	438 cm (94–1089)	29.5 cm (11.5–46)	5
	Wood	128 cm (0–274)	100 cm (12.5–411)	9
	Rock	79.8 cm (0–530)	30.4 cm (3–101)	53
	All substates	106.0 cm (0–1089)	35.8 cm (3–411)	71
Natural	Rock	56.1 cm (0–620)	38.6 cm (5–83)	89

*Note:* Nests in anthropogenic cavities with rock substrate included nests in human‐made rock piles, gabions, or revetments. Nests in buildings were in vents, openings in brick, wood, or plastic siding. Nests in metal included nests in pipes, in shipping containers, and on unused machinery. Nests in wood were on the ground under discarded plywood sheeting, in wood piles, or in one case, in a wooden shed with no door.

**FIGURE 3:**
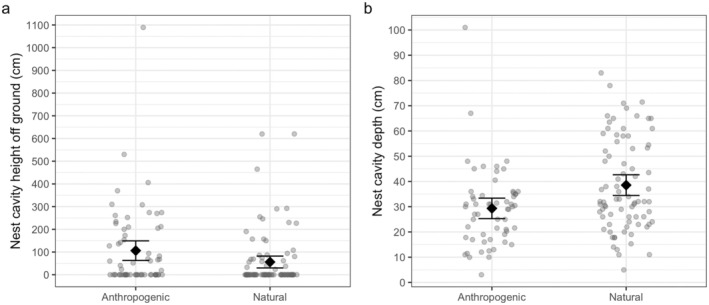
(a) Height (distance from ground to lower edge of nest cavity, *n* = 152) and (b) depth (distance from entrance of nest cavity to rim of nest, *n* = 138) of anthropogenic and natural nest cavities in Iqaluit, NU. Diamonds show mean and whiskers extend to 95% confidence limits around the mean. Individual data points are jittered to remove overlap. Note that depth for one nest in a wooden shed was omitted in (b) for clarity; this nest was accessed through the open door of the shed resulting in nest depth of > 4 m.

Nests in natural cavities were found more often at ground level (68% of natural nests, Figure 3) whereas only 49% of nests in artificial substrate were at ground level. Nest height for both cavity types was right skewed due to many nests at or near ground level, therefore we used a Wilcoxon rank‐sum test to compare nest heights by type. We found that anthropogenic cavities were significantly higher than natural cavities (*W* = 3422.5, *p* = 0.012; Table 1). Of nests that were off the ground, the mean height for artificial cavities was 212 ± 34.5 cm (mean ± standard error, range: 19–1089 cm), and for natural cavities was 174 ± 30.9 cm (range: 24–620 cm). The proportion of nests on the ground was significantly higher for natural nest cavities ($\chi^{2}$ = 4.25, df = 1, *p* = 0.039). Nest depth, that is, the depth of the edge of the nest from the cavity entrance, was not significantly different between cavity types (mean 35.8 ± 6.17 cm in artificial, range: 3–411 cm, and mean 38.6 ± 1.94 cm in natural nests, range: 5–83 cm; *t* = −0.38, df = 68.78, *p* = 0.70). We examined the bearing of the nest from the cavity entrance facing outwards and tested for differences between natural and artificial nest cavities. Nest bearing was highly dispersed (global concentration parameter < 2), violating assumptions of the Watson Williams test for homogeneity of means (Agostinelli and Lund 2024), therefore we used a nonparametric circular test, the Watson Wheeler test, which found no significant difference in bearing between artificial and natural nest cavities (*W* = 1.85, df = 2, *p* = 0.40). Nests in both cavity types tended to face southwest (195° average for artificial cavities, 224° for natural cavities).

## 4 Discussion

Snow Buntings nested in a variety of human‐created structures in the city of Iqaluit, Nunavut. Despite the availability of nesting sites in buildings, wood, and metal substrate, 77% of anthropogenic nest cavities were in rock, the same substrate as all natural nests in the city. Nests in human‐built structures in Iqaluit, regardless of substrate, were more likely to be above ground level compared to nests in natural cavities. Nests above the snow level are available earlier in the nesting season, and may be less accessible to ground‐based predators, such as rodents (lemming *Lemmus lemmus* ), mustelids (ermine *Mustela erminia*), dogs (*Canis domesticus*) or cats (*Catus domesticus*). Nests above ground may also have an advantage during extreme weather, when rain can flood ground‐level nests and cause chick and egg mortality. Rainfall is expected to increase in the Arctic under climate change models (Bintanja and Andry 2017), and ground nests in rock are particularly vulnerable as excess water run‐off cannot be absorbed. Nestling mortality of rock‐ledge nesting Peregrine Falcons ( *Falco peregrinus* ) in the Canadian Arctic was higher with increasing rainfall, an effect which could be mitigated by providing elevated wooden nest boxes (Anctil et al. 2014).

About 10% of all nests we found were in cavities made of anthropogenic materials, such as wood, metal, or mixed material (buildings). For passerines nesting in European forests, human‐provided nest boxes of wood or woodcrete had relatively unstable nest microclimates compared with natural nest cavities in trees and tended to have higher temperature maxima across all stages of the nesting cycle (Sudyka et al. 2023). Summer temperatures in Iqaluit are relatively cool (average June–July high temperatures 5°C–12°C with zero humidity; Weatherspark.com 2025); however, Snow Buntings are highly adapted to cold temperatures and can be behaviourally constrained at temperatures higher than 11.7°C (O'Connor et al. 2022). Nesting in anthropogenic materials that increase nest temperatures could create heat stress for Snow Buntings.

Nest predation rates could also differ in anthropogenic cavities; Pied Flycatchers ( *Ficedula hypoleuca* ) nesting in boxes showed advanced lay dates compared to those in natural cavities, but also had higher nest predation rates (Dorota 2004), as did Collared Flycatchers ( *Ficedula albicollis* , Mitrus 2003). Snow Buntings do use nest boxes in other parts of their range, for example, in Alaska, USA (Keim 2022), Svalbard, Norway, and in Russia (Montgomerie and Lyon 2020). There is some evidence of differences in Snow Bunting chick weight and growth rate between natural and artificial nest cavities (Hilmarsen 2020), but overall effects on reproductive success or survival are not known. Given the lack of information on bunting population trends, understanding the impacts of both urbanization and climate change on productivity is important. Most studies of artificial nest cavity use come from southern latitudes, where nests in boxes of wood are compared with natural cavities in trees (e.g., Sudyka et al. 2023) and Snow Buntings in the urban Arctic may show differences in their response to the increasing availability of anthropogenic nest cavities.

## Author Contributions

**Samuelle Simard‐Provençal:** data curation (equal), investigation (equal), methodology (equal), writing – review and editing (equal). **Patricia Rokitnicki:** data curation (equal), investigation (equal), methodology (equal), writing – review and editing (equal). **Rebecca Golat:** investigation (equal), methodology (equal), writing – review and editing (equal). **François Vézina:** conceptualization (equal), funding acquisition (equal), investigation (equal), methodology (equal), project administration (equal), resources (equal), supervision (equal), writing – review and editing (equal). **Oliver P. Love:** conceptualization (equal), data curation (equal), funding acquisition (lead), investigation (equal), methodology (equal), project administration (lead), resources (lead), supervision (lead), writing – review and editing (equal). **Emily A. McKinnon:** conceptualization (equal), data curation (equal), formal analysis (equal), investigation (equal), methodology (equal), project administration (equal), writing – original draft (lead), writing – review and editing (equal).

## Conflicts of Interest

The authors declare no conflicts of interest.

## Data Availability

Data and R scripts used in this study are available on Figshare: https://figshare.com/s/f69bda60e6781bef3564.
